# Blood-borne miRNA profile-based diagnostic classifier for lung adenocarcinoma

**DOI:** 10.1038/srep31389

**Published:** 2016-08-10

**Authors:** Mei Chee Tai, Kiyoshi Yanagisawa, Masahiro Nakatochi, Naoe Hotta, Yasuyuki Hosono, Koji Kawaguchi, Mariko Naito, Hiroyuki Taniguchi, Kenji Wakai, Kohei Yokoi, Takashi Takahashi

**Affiliations:** 1 Division of Molecular Carcinogenesis, Center for Neurological Diseases and Cancer, Nagoya University Graduate School of Medicine, Nagoya, Japan; 2 Center for Advanced Medicine and Clinical Research, Nagoya University Hospital, Nagoya, Japan; 3 Department of Thoracic Surgery, Nagoya University Graduate School of Medicine, Nagoya, Japan; 4 Department of Preventive Medicine, Nagoya University Graduate School of Medicine, Nagoya, Japan; 5 Department of Respiratory Medicine and Allergy, Tosei General Hospital, Seto, Japan

## Abstract

Accumulated evidence indicates that various types of miRNA are aberrantly expressed in lung cancer and secreted into the bloodstream. For this study, we constructed a serum diagnostic classifier based on detailed bioinformatics analysis of miRNA profiles from a training cohort of 143 lung adenocarcinoma patients and 49 healthy subjects, resulting in a 20 miRNA-based classifier. Validation performed with an independent cohort of samples from lung adenocarcinoma patients (n = 110), healthy subjects (n = 52), and benign pulmonary disease patients (n = 47) showed a sensitivity of 89.1% and specificity of 94.9%, with an area under the curve value of 0.958. Notably, 90.8% of Stage I lung adenocarcinoma cases were correctly diagnosed. Interestingly, this classifier also detected squamous and large cell lung carcinoma cases at relatively high rates (70.4% and 70.0%, respectively), which appears to be consistent with organ site-dependent miRNA expression in cancer tissues. In contrast, we observed significantly lower rates (0–35%) using samples from 96 cases of cancer in other major organs, with breast cancer the lowest. These findings warrant a future study to realize its clinical application as a part of diagnostic procedures for lung cancers, for which early detection and surgical removal is presently the only hope for eventual cure.

Lung cancer is the leading cause of cancer-related mortality, with adenocarcinoma the most prevalent among the four major subtypes, though accumulated evidence indicates marked distinctions among adenocarcinomas in terms of genetic and epigenetic alterations^1^,^2^. Early-stage lung adenocarcinomas are mostly asymptomatic and often diagnosed at a later clinical stage, making surgical resection impossible as a curative strategy^3^. At present, detection of lung adenocarcinoma relies to a large extent on imaging procedures such as chest X-rays and CT scans, and only a few blood markers such as carcinoembryonic antigen have been developed for diagnosis of lung adenocarcinoma, while their sensitivity and specificity are unsatisfactory for routine clinical use^3^. Therefore, development of a sensitive and reliable blood biomarker that can be obtained in an inherently minimally invasive manner is highly anticipated.

microRNAs (miRNAs) are small non-coding single-stranded RNAs that regulate gene expression by binding to 3′ untranslated regions of their target genes. Each miRNA can affect up to hundreds of target genes, thereby influencing multiple oncogenic and/or tumor suppressive pathways^4^,^5^. Following our initial discovery of down-regulation of let-7 6, a number of oncogenic and tumor suppressive miRNAs have been reported to exhibit altered expression in lung cancer tumor tissues^7^,^8^,^9^,^10^. In addition, differences in regard to blood-borne circulating miRNAs have been reported between lung cancer patients and disease-free individuals^11^, which appears to reflect the fact that miRNAs can be stably incorporated into micro-vesicles^12^. Considering the markedly distinct molecular pathogenesis, it is speculated that lung adenocarcinoma patients may bear distinctive profiles of circulating miRNAs in the bloodstream. However, the vast majority of previous reports of miRNA-based serum/plasma biomarkers dealt with non-small cell lung cancer (NSCLC) in only a broad manner. In contrast, very few studies have aimed at developing an miRNA biomarker specifically for lung adenocarcinoma, while even fewer have attempted to construct diagnostic classifiers based on a blood-borne miRNA profile with use of an independent validation cohort including other types of cancers^13^,^14^,^15^,^16^,^17^.

In the present study, we attempted to establish an miRNA profile-based diagnostic method by use of a training cohort of serum samples from lung adenocarcinoma patients. The resultant classifier was then validated with an independent validation cohort. We report here our results showing successful construction and validation of a serum miRNA profile-based classifier for diagnosis of lung adenocarcinoma.

## Results

### Search for miRNAs useful as biomarkers or internal control

**Figure 1:**
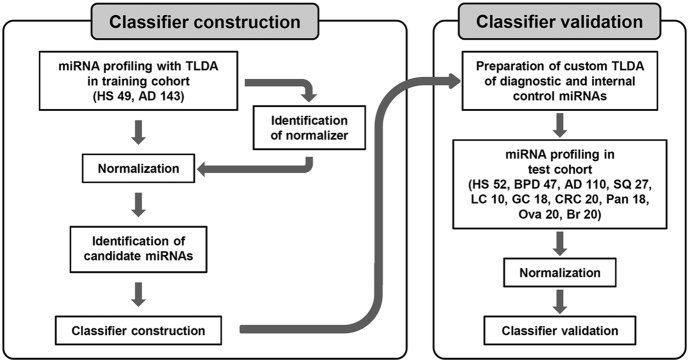
Schematic diagram of our training-validation strategy for constructing a diagnostic classifier for lung adenocarcinoma. We used 192 samples from 143 patients with lung adenocarcinoma (AD) and 49 from healthy subjects (HS) as a training cohort, while the independent validation cohort was comprised of 342 samples, including 110 AD, 27 squamous cell lung carcinoma (SQ), 10 large cell lung carcinoma (LC), 18 gastric cancer (GC), 20 colorectal cancer (CRC), 18 pancreatic cancer (Pan), 20 ovarian cancer (Ova), and 20 breast cancer (Br) cases, as well as 52 HS and 47 benign pulmonary disease (BPD) samples. TLDA, TaqMan low density array for miRNA expression analysis.

The present study was conducted using an overall scheme with two clearly separated stages; construction of a diagnostic classifier based on analysis of a training cohort using ready-made TaqMan Human MicroRNA Arrays (cards A and B) containing 768 miRNAs, and validation of the resultant classifier with an independent cohort and a custom-made TaqMan Human MicroRNA array, along with a completely independent set of blood samples from lung adenocarcinoma patients as well as those with other types of cancers (Fig. 1).

**Figure 2:**
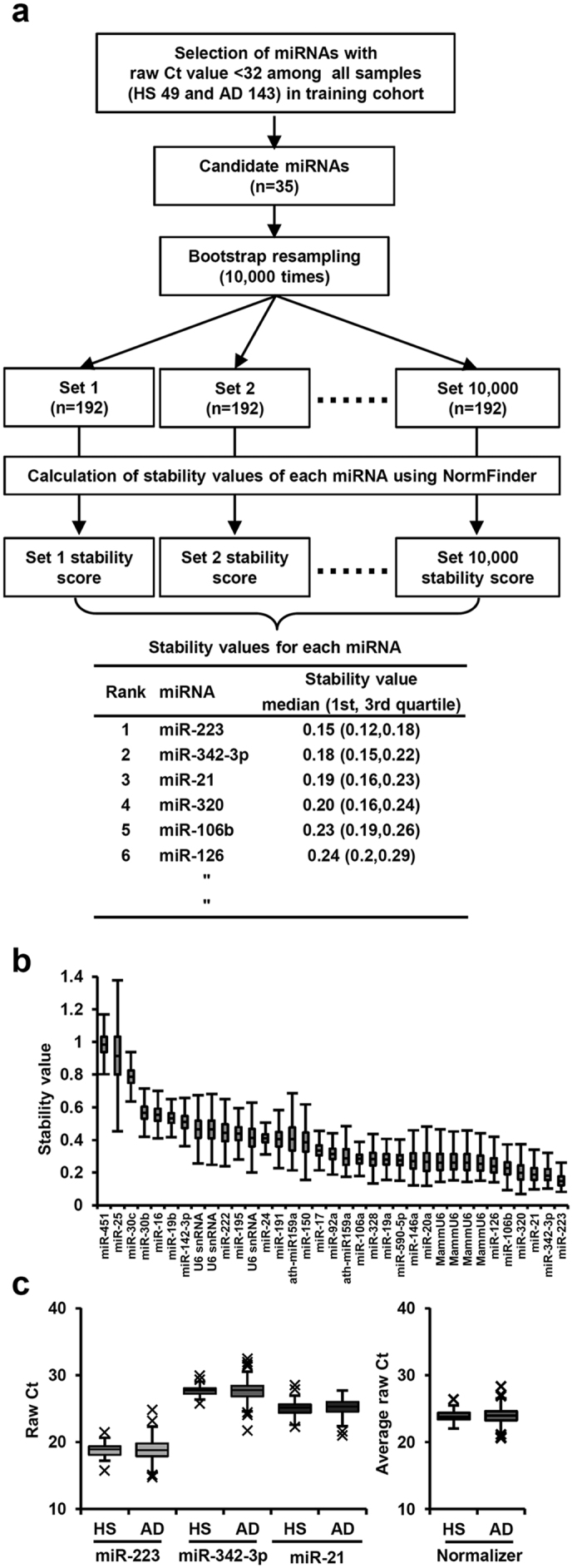
Search for miRNAs stably present in blood samples and thus suitable for normalization. (**a**) Strategy for selecting stable present miRNAs using NormFinder with 10,000-times bootstrap resampling of the training cohort. (**b**) Stability values for each candidate for internal control miRNA. (**c**) Box plot analysis of Ct values of top 3 miRNAs and the resultant normalizer. HS, healthy subjects; AD, lung adenocarcinoma.

Since no normalizer for analysis of blood-borne miRNAs has been definitively established, we first searched for miRNAs that could be used as an internal control to normalize the quality and quantity of a blood sample. Using 192 training cohort samples, we detected 35 miRNAs (Ct < 32) that were considered to be normalizer candidates, and subjected them to bootstrap resampling 10,000 times and subsequent statistical analysis using NormFinder (Fig. 2a). The candidate miRNAs were rank-ordered according to their median stability values (Fig. 2b, Supplementary Table S1). We observed small variations among the samples for the top 3 miRNAs, which were miR-21, miR-223, and miR-342-3p (Fig. 2c, left panel), and thus defined their average of Ct values as the normalizer value (Fig. 2c, right panel).

**Figure 3:**
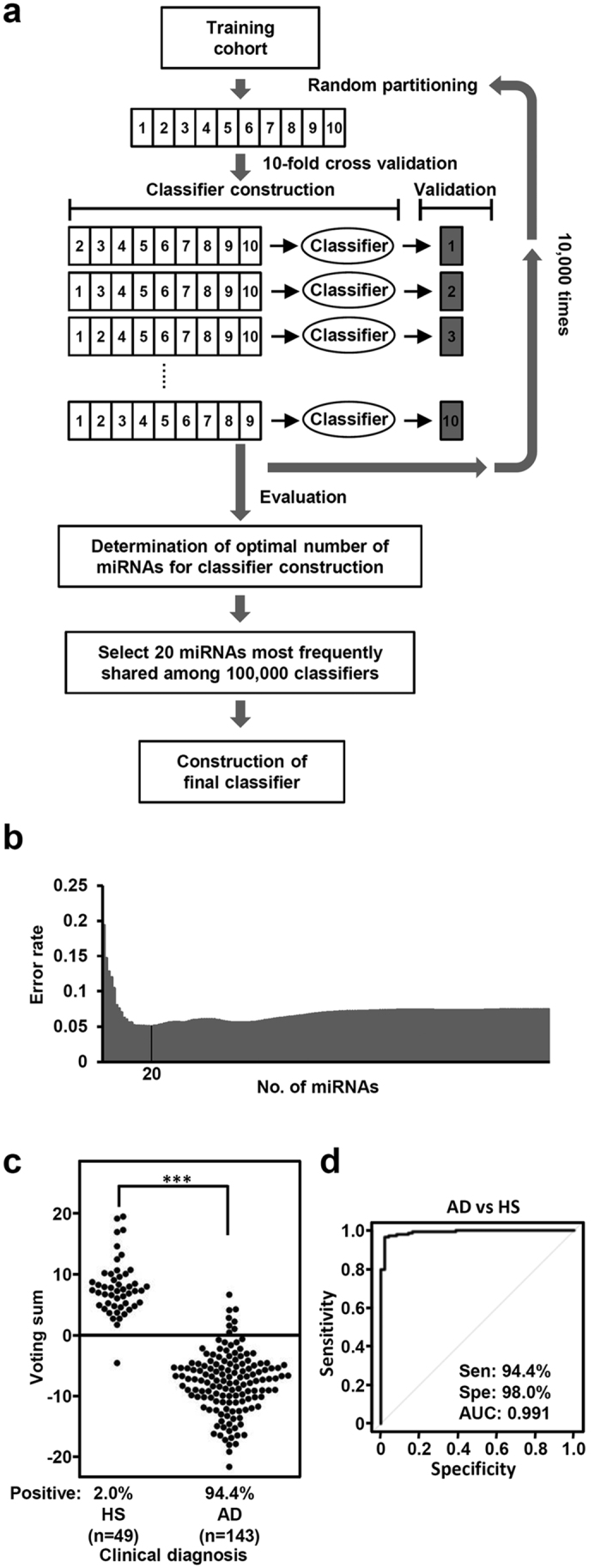
Construction of weighted voting diagnostic classifier using 10-fold cross validation and 10,000-times random partitioning of the training cohort. (**a**) Schematic diagram showing construction of blood-borne miRNA profile-based diagnosis classifier. (**b**) Results of our search for the optimum number of miRNAs for diagnosis of lung adenocarcinoma, which showed 20 miRNAs with the lowest error rate. (**c**) Assessment of the 20 miRNA-based weighted voting classifier for diagnosis of lung adenocarcinoma. (**d**) ROC curve analysis of the diagnostic classifier, which showed an AUC value of 0.991. HS, healthy subjects; AD, lung adenocarcinoma; Sen, sensitivity; Spe, specificity.

Next, we employed a weighted-voting algorithm, a well-established method for supervised machine learning, in which each weighted value was calculated based on the signal-to-noise (S2N) ratio. We used 10-fold cross-validation with random partitioning performed 10,000 times during this process in order to minimize over-fitting to the training cohort and construct a generally applicable classifier (Fig. 3a). Our findings revealed 20 miRNAs that resulted in the lowest number of misclassifications among the 100,000 cross-validations (Fig. 3b). These top 20 miRNAs, which were most commonly shared among the 100,000 classifiers constructed each time, were consequently selected to construct a final version of the classifier (Table 1). In subsequent analysis of serum samples from 143 lung adenocarcinoma patients and 49 disease-free individuals that comprised the training set, the final classifier yielded a sensitivity of 94.4% and specificity of 98.0%, as well as an overall classification accuracy of 95.3% (Fig. 3c). Receiver operating characteristic (ROC) analysis showed a very high value of 0.991 for the area under the curve (AUC) of the 20 miRNA-based classifier (Fig. 3d).

### Validation of final classifier using independent validation samples

**Figure 4:**
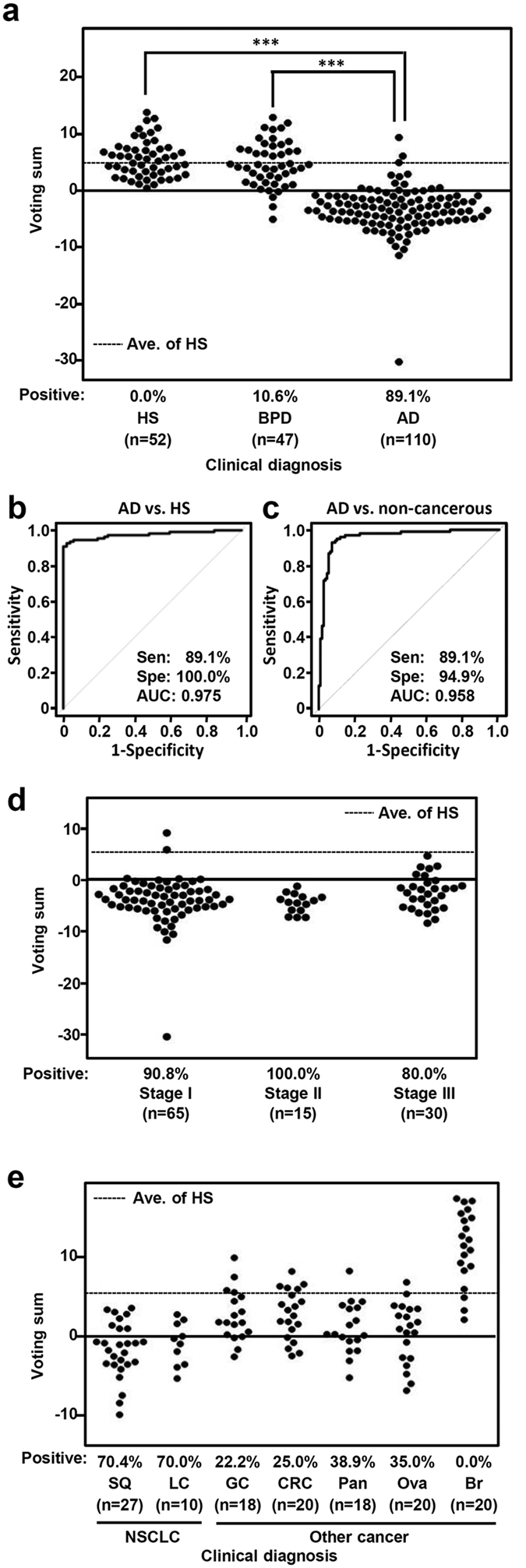
Validation analysis of resultant diagnostic classifier using independent cohort of samples from healthy subjects (HS) and patients with lung adenocarcinoma (AD), as well as patients with benign pulmonary disease (BPD) and various other types of cancer. (**a**) Assessment of diagnostic classifier using an independent cohort. Ave. of HS, average of voting sum in HS. (**b**) ROC curve for validation cohort consisting of samples from 52 healthy subjects and 110 patients with lung adenocarcinoma. (**c**) ROC curve for discriminating patients with lung adenocarcinoma from either healthy subjects and patients with pulmonary lung disease. (**d**) Assessment of diagnostic classifier according to disease stage in lung adenocarcinoma cases. (**e**) Assessment of diagnostic classifier in other types of cancer.

In order to validate the robustness of the diagnostic classifier for lung adenocarcinoma, an independent test set consisting of blood samples obtained from 110 adenocarcinoma patients and 52 disease-free subjects were analyzed using a custom made TaqMan Human MicroRNA array harboring 20 diagnostic and 3 internal control miRNAs. In addition, blood samples from 47 patients with benign pulmonary disease, including 35 interstitial pneumonia, 4 bacterial pneumonia, 4 aspergilloma, 3 pulmonary tuberculosis, and 1 pulmonary sequestration cases, were examined to investigate whether the present miRNA-based classifier is capable of discriminating lung adenocarcinoma from benign pulmonary disease. Our results showed that 89.1% (98 of 110) of the lung adenocarcinoma patients were correctly classified into the positive diagnosis group, while all 52 (100%) disease-free individuals and 42 of 47 (89.4%) patients with benign pulmonary disease were appropriately defined as negative for lung adenocarcinoma (Fig. 4a). ROC analysis showed that the AUC value for discrimination between lung adenocarcinoma and disease-free individuals was 0.975 (Fig. 4b), while an AUC value of 0.958 was attained in analysis of lung adenocarcinoma *versus* non-cancerous subjects (Fig. 4c). It was also notable that the present diagnostic classifier was able to correctly diagnose early stage lung adenocarcinoma cases, as 92.5% (74 of 80) in stages I and II were positive (Fig. 4d). Together, these findings demonstrate that our novel 20 miRNA-based classifier is a useful blood-borne diagnostic method for detection of lung adenocarcinoma.

We then determined what proportions of other types of NSCLC (n = 37) and cancer occurring outside the lung (n = 96) would yield positive results with the present diagnostic classifier. Interestingly, we found that both squamous cell and large cell carcinomas of the lung were positive in relatively high proportions (70.4% and 70.0%, respectively), suggesting considerable usefulness of the classifier for diagnosis of NSCLC in addition to lung adenocarcinoma. In contrast, cancer occurring in other organ sites exhibited much lower positivity, with 22.2% of gastric cancer, 25.0% of colorectal cancer, 38.9% of pancreatic cancer, 35.0% of ovarian cancer, and 0% of breast cancer cases returning positive results (Fig. 4e). These findings indicate organ site-dependent specificity in terms of detection using the present diagnostic classifier.

## Discussion

In the present study, we constructed a diagnostic classifier based on the results of miRNA profiling analysis using serum samples from lung adenocarcinoma patients and demonstrated that the presence of lung adenocarcinoma can be detected with only a small amount of serum. Our novel 20 miRNA-based classifier showed a sensitivity of 89.1% and specificity of 94.9% to discriminate lung adenocarcinoma patients from disease-free individuals as well as patients with benign pulmonary diseases, with a remarkably high AUC value of 0.958. It is also notable that 90.8% of Stage I lung adenocarcinoma cases were correctly diagnosed. In addition, only 5 of 47 patients with benign pulmonary disease were falsely diagnosed as having a lung adenocarcinoma, also supporting the usefulness of our novel present classifier as an adjunct means for discriminating lung adenocarcinoma from benign pulmonary disease and other types of cancer.

Previous studies have attempted to develop diagnostic methods for lung cancer using detection of blood-borne miRNAs. However, those focused more broadly on NSCLC as a whole, while successful establishment of a serum miRNA profile-based diagnostic classifier specifically for lung adenocarcinoma has been scarcely reported, with, to the best of our knowledge, the previous study by Bianchi *et al*. presenting the only such findings^13^. In that study, they constructed a 34 miRNA-based classifier using serum obtained from 25 patients with lung adenocarcinoma and 39 disease-free subjects, then validation was done with an independent test cohort consisting of 30 disease-free subjects, and 22 adenocarcinoma and 12 squamous cell carcinoma patients, resulting in an AUC value of 0.85 for diagnosis of lung adenocarcinoma.

The present classifier was constructed and validated with larger sample sizes, and showed considerably higher performance. In addition, we evaluated our classifier by using 96 cases of a wide range of cancer occurring in other organ sites, in contrast to the inclusion of only 18 breast cancer cases as the sole other cancer type in that previous study. We also observed a significantly lower false positive rate (23.4%) in the panel of 96 other cancer types, with breast cancer showing the lowest rate. The distinct detection rates dependent on the originating organ site observed in the present study may be associated with the presence of distinct miRNA expression profiles in various types of human cancer tissues^18^,^19^. Along this line, it is also interesting to note that in addition to the lung adenocarcinomas cases, those with squamous cell or large cell carcinoma also exhibited high rates of positive diagnosis, even though the present classifier was constructed using a training set solely consisting of lung adenocarcinomas samples.

A few other studies have aimed at constructing a general diagnostic classifier for NSCLC. Chen *et al*. reported a 10 miRNA-based signature for diagnosis of NSCLC with a large cohort comprised of 200 cases and 110 controls for training, as well as the same numbers for validation, which attained an AUC value of 0.972 in the validation step^16^. Boeri *et al*. identified a signature comprised of 16 sets of expression ratios involving 13 miRNAs based on a training set with individual blood samples obtained from 19 NSCLC patients and 5 pools of normal sera^14^. Their ratio-based signature was then validated using an independent test cohort of 22 NSCLC samples and 10 pools from normal subjects, which showed an AUC value of 0.88. That research group also conducted a subsequent study of a modified version of their classifier consisting of 27 samples from 18 miRNAs with a larger cohort consisting 69 lung cancer patients and 870 disease-free subjects^17^.

The present 20 miRNA-based diagnostic classifier shares miR-19b, miR-24, miR-126-5p, miR-142-5p, and miR-30c as diagnostic miRNAs with other presented classifiers^13^,^14^,^16^. It should be noted that suitable reference genes for relative quantification of miRNA levels in serum have not been established, despite the fundamental importance of such quantification for reliable and stable data acquisition. In this regard, combined use of miR-21, miR-223, and miR-342-3p was found to be useful for normalization of RNA in each blood sample. Although our reference miRNA set is distinct from those proposed by others, candidates selected in this study included 2 miRNAs reported by Silva *et al*.^15^ and 4 of 6 miRNAs reported by Bianchi *et al*.^13^, albeit at lower levels of stability.

In conclusion, the present study successfully established and validated a diagnostic classifier using serum samples based on a signature consisting of 20 miRNAs. Carefully designed and detailed bioinformatics analysis allowed us to construct this classifier, with very high levels of sensitivity and specificity shown by reliable data acquired with use of a normalizer. To date, there are very few clinically useful biomarkers for lung adenocarcinoma, and their sensitivity and specificity are far from adequate. The present results along with accumulating evidence suggest that measurement of signatures consisting of blood-borne miRNAs will become an important component of diagnostic procedures, including potential use to reduce the high false-positive rate in low-dose CT screening for this devastating disease, for which early detection and surgical removal are currently the only means to provide hope for eventual cure.

## Methods

### Patients and specimens

Serum samples were provided by a total of 542 individuals, which included 102 disease-free subjects and 393 patients with cancer (lung adenocarcinoma 260, squamous cell lung carcinoma 27, large cell lung carcinoma 10, gastric cancer 18, colorectal cancer 20, pancreatic cancer 18, ovarian cancer 20, breast cancer 20), as well as 47 patients with benign pulmonary disease (interstitial pneumonia 35, bacterial pneumonia 4, aspergilloma 4, pulmonary tuberculosis 3, pulmonary sequestration 1). The disease-free subjects were from a cohort enrolled in an epidemiologic study conducted by the Department of Preventive Medicine, Nagoya University, Nagoya, Japan. Lung cancer patients being treated at Nagoya University Hospital, Nagoya, Japan, as well as patients with benign pulmonary diseases treated at Nagoya University Hospital or Tosei General Hospital, Seto, Japan, donated blood samples. Serum samples from patients with other types of cancers were collected by the Kanagawa Cancer Research and Information Association. Samples from the 102 disease-free subjects and 260 patients with adenocarcinoma were first separated into 2 groups based on age-, sex-, and disease stage-matched sample sets, i.e., training and validation sets. The training set eventually consisted of samples from 49 disease-free subjects and 143 lung adenocarcinoma patients, while the validation set was composed of 52 and 110, respectively. There were no statistically significant differences in regard to clinicopathologic features between the training and validation sets (Table 2). Additional serum samples from patients with other types of cancer as well as benign pulmonary disease were also used for validation. All the experimental protocols were approved by the institutional review boards of Nagoya University Graduate School of Medicine as well as the Kanagawa Cancer Research and Information Association in accordance with the approved guidelines, and written informed consent from all subjects had been obtained.

### Total RNA isolation

Total RNA was isolated from 400-μL serum samples using an miRVana PARIS kit (Ambion) according to the manufacturer’s protocol for total RNA isolation from liquid samples, with a minor modification. Briefly, synthetic RNA of ath-miR159a (UUUGGAUUGAAGGGAGCUCUA) was added to each sample as a spike control for evaluation of RNA extraction. The resultant total RNA concentrations were quantified by use of a NanoDrop 2000 spectrophotometer (Thermo Scientific).

### Determination of microRNA profiles

The presence of 754 human miRNAs along with the ath-miR-159a, U6, and RNU genes was profiled in each serum sample using a TaqMan Human MicroRNA array Card (A, v2.0, and B, v3.0, both from Life Technologies), according to the manufacturer’s instructions. Briefly, 6 μg of total RNA (3 μl per reaction) was reverse transcribed using a TaqMan miRNA Reverse Transcription Kit (Life Technologies) in combination with the stem-loop Megaplex primers pool set A or B in a total volume of 7.5 μl. Megaplex reverse transcription products were further pre-amplified using TaqMan PreAmp Master Mix and Megaplex PreAmp primers (Life Technologies), which were then subjected to real-time PCR analysis using TaqMan Human MicroRNA arrays and an ABI Prism 7900HT Sequence Detection System (Life Technologies). Raw Ct values were calculated using RQ manager software v1.2.1 (Life Technologies). Validation of the constructed diagnostic classifier was conducted in the same manner using a custom-made TaqMan Human MicroRNA Array, and RT and PreAmp primers (Life Technologies), after calibration with use of 22 samples from the training set, which had been analyzed using the above-mentioned commercially available set.

### Bioinformatics and statistical analyses

In the training cohort, which consisted of samples from 150 adenocarcinoma patients and 50 healthy subjects, the presence of 754 human miRNAs was determined using a TaqMan Human MicroRNA array Card. For quality control, 3 samples were removed because of an undetectable spike in miRNA of ath-miR159a. Then, the 25th percentile of Ct values for 349 probes, which had a Ct value <32 in at least 1 sample, was calculated for each sample, with 5 samples whose 25th percentile value was ≥ 32 excluded (Supplementary Fig. 1). As a result, a total of 192 samples (143 adenocarcinoma patients, 49 healthy subjects) remained as the training cohort for further analysis. We then searched for miRNAs suitable for use as an internal control. First, 35 probes detected (Ct < 32) in all 192 samples of the training cohort were selected as candidates for use as a normalizer then subjected to calculations of stability value for each miRNA as a normalizer using NormFinder for R^20^, with bootstrap resampling performed 10,000 times (Fig. 2a). Next, the candidate miRNAs were rank-ordered according to their median stability value obtained in bootstrap resampling. Finally, we selected the top 3 miRNAs to define a normalizer set, and the mean value of their Ct values was used for normalization.

In all samples subjected to measurement using a TaqMan Human MicroRNA array Card, expression levels were determined based on the ΔΔCt method^21^. To calculate expression levels, we used a normalizer defined as above and mean values of 22 calibrator samples for each miRNA. Those ΔΔCt values were transformed into z-scores based on the mean and SD values across all 192 samples in the training cohort. Finally, the z-scores were used for construction of the classifier. One hundred seventy-eight probes, detected (Ct < 32) in at least 10% of the adenocarcinoma patient samples in the training cohort, were considered to be candidate miRNAs for construction of the classifier. We then used a weighted voting algorithm, in which each weighted value was calculated as the signal-to-noise ratio, according to the method that we previously described in detail, with a slight modification^22^. Briefly, 10-fold cross-validation with random partitioning performed 10,000 times was carried out during this process in order to minimize over-fitting to the training cohort and construct a generally applicable classifier (Fig. 3a). The model constructed in the training dataset was assessed by applying it to validation datasets of other types of lung cancer as well as various types of other cancer.

### Other biostatistical analyses

Analysis of variance, Student’s t-test, and Fisher’s exact test were used to assess mean values among the groups. Receiver operating characteristics (ROC) analysis was performed to evaluate the performance of the constructed model using pROC of the R package^23^. All statistical analyses was performed with R software version 3.2 (www.r-project.org) and the two-sided significance level was set at P < 0.05.

## Additional Information

**How to cite this article**: Tai, M. C. *et al*. Blood-borne miRNA profile-based diagnostic classifier for lung adenocarcinoma. *Sci. Rep*. **6**, 31389; doi: 10.1038/srep31389 (2016).

## Supplementary Material

Supplementary Information

## Figures and Tables

**Table 1:** miRNAs included in 20 miRNA-based diagnostic classifier.

miRNAs	Average rank in 100,000 independent models	Number of times included in 100,000 independent modeling^a^
miR-451	1.06	100,000
miR-1290	2.83	100,000
miR-636	3.65	100,000
miR-30c	4.61	100,000
miR-22-3p	5.35	100,000
miR-19b	5.50	100,000
miR-486-5p	7.96	100,000
miR-20b	8.39	100,000
miR-93	9.07	100,000
miR-34b	9.55	99,999
miR-185	12.13	99,966
miR-126-5p	13.71	99,516
miR-93-3p	11.95	99,426
miR-1274a	14.99	99,301
miR-142-5p	16.35	87,826
miR-628-5p	17.22	80,362
miR-486-3p	17.13	79,091
miR-425	17.73	73,983
miR-645	19.22	66,461
miR-24	19.04	65,809

^a^100,000 models of diagnostic classifier were constructed through 10-fold validation with 10,000 repartitioning of the training cohort.

**Table 2:** Clinicopathologic characteristics of training and test cohorts.

Variable	Lung adenocarcinoma patients			Healthy subjects		
	Training	Test	*p*	Training	Test	*p*
No. of samples	143	110		49	52	
Age at diagnosis (years)^c^	65.9 ± 9.8^a^	65.0 ± 9.9	0.494^b^	66.3 ± 8.9	65.7 ± 9.7	0.774^b^
**Gender**						
Male	76	62	0.703^c^	28	30	1.000^c^
Female	67	48		21	22	
**pStage**						
I	80	65	0.273^c^			
II	21	15				
III	37	30				
IV	5	0				
Smoking history^d^	728.2 ± 770.2 (n = 116)	634.4 ± 777.0 (n = 70)	0.424^b^	320.5 ± 578.3 (n = 48)	272.6 ± 459.6 (n = 51)	0.648^b^

^a^Average age ± S.D.

^b^Student’s t-test.

^c^Fisher’s exact test.

^d^Brinkman Index.
