# Supplementary material for: Blood-borne miRNA profile-based diagnostic classifier for lung adenocarcinoma
Source: Sci Rep. 2016 Aug 10;6:31389. doi: 10.1038/srep31389 (PMC4979017; doi:10.1038/srep31389)
Supplement: Supplementary Information [file srep31389-s1.pdf]

Blood-borne miRNA profile-based diagnostic classifier for lung adenocarcinoma

Mei Chee Tai, Kiyoshi Yanagisawa, Masahiro Nakatochi, Naoe Hotta, Yasuyuki Hosono, Koji Kawaguchi, Mariko Naito, Hiroyuki Taniguchi, Kenji Wakai, Kohei Yokoi, Takashi Takahashi

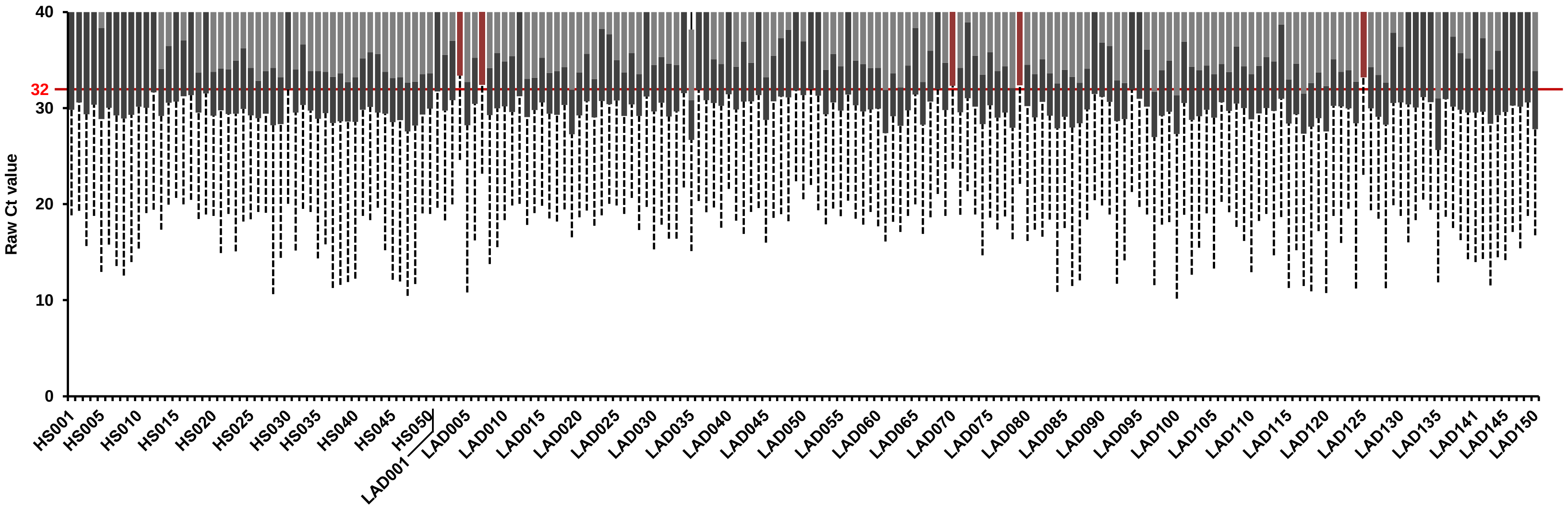

Supplementary Figure S1. Box plot analysis of raw Ct values for training cohort consisting of samples from 49 healthy subjects (HS) and 148 patients with lung adenocarcinoma (AD), which were initially subjected to TLDA analysis. Data from 5 cases indicated in red were excluded from the subsequent normalizer identification and classifier construction, because of possible poor quality RNA.

Supplementary Table S1. Stability value for each candidate for internal control miRNA

Rank	miRNAs	Stability value Median (1st, 3rd quartile)
1	miR-223	0.15 (0.12, 0.18)
2	miR-342-3p	0.18 (0.15, 0.22)
3	miR-21	0.19 (0.16, 0.23)
4	miR-320	0.20 (0.16, 0.24)
5	miR-106b	0.23 (0.19, 0.26)
6	miR-126	0.24 (0.20, 0.29)
7	MammU6	0.25 (0.21, 0.30)
8	MammU6	0.26 (0.22, 0.31)
9	MammU6	0.26 (0.22, 0.31)
10	MammU6	0.26 (0.22, 0.32)
11	miR-20a	0.26 (0.21, 0.32)
12	miR-146a	0.27 (0.22, 0.32)
13	miR-590-5p	0.28 (0.25, 0.31)
14	miR-19a	0.28 (0.25, 0.31)
15	miR-328	0.28 (0.24, 0.32)
16	miR-106a	0.28 (0.26, 0.31)
17	ath-miR159a	0.29 (0.25, 0.34)
18	miR-92a	0.31 (0.28, 0.35)
19	miR-17	0.34 (0.31, 0.37)
20	miR-150	0.39 (0.33, 0.44)
21	ath-miR159a	0.40 (0.34, 0.48)
22	miR-191	0.40 (0.36, 0.45)
23	miR-24	0.41 (0.38, 0.43)
24	U6 snRNA	0.41 (0.36, 0.47)
25	miR-195	0.44 (0.40, 0.48)
26	miR-222	0.44 (0.39, 0.50)
27	U6 snRNA	0.46 (0.41, 0.52)
28	U6 snRNA	0.47 (0.41, 0.52)
29	miR-142-3p	0.51 (0.47, 0.55)
30	miR-19b	0.53 (0.50, 0.56)
31	miR-16	0.55 (0.52, 0.59)
32	miR-30b	0.57 (0.53, 0.60)
33	miR-30c	0.79 (0.75, 0.82)
34	miR-25	0.92 (0.80, 1.03)
35	miR-451	0.98 (0.94, 1.03)
